# Supplementary material for: ARFID Genes and Environment (ARFID-GEN): study protocol
Source: BMC Psychiatry. 2023 Nov 21;23:863. doi: 10.1186/s12888-023-05266-x (PMC10664384; doi:10.1186/s12888-023-05266-x)
Supplement: Supplementary file 1 — Additional file 1. [file 12888_2023_5266_MOESM1_ESM.docx]

**Supplement to:**

**ARFID GENES and ENVIRONMENT (ARFID-GEN): Study Protocol**

Bulik et al.

**TABLE OF CONTENTS**

Self- and parent/guardian report measures……………………………………………………….……………Page 2

Aim 2 detailed methods (Within disorder ARFID GWAS) ………………………………….………..……Page 9

Aim 3 detailed methods (Genetic relation of ARFID to other eating disorders)………………..Page 17

**Self- and Parent/Guardian Report Measures**

***ARFID Symptoms and Diagnosis***

Nine Item Avoidant/Restrictive Food Intake Disorder Screen (NIAS) ([1](#_ENREF_1)). A brief questionnaire with three subscale scores (i.e., picky eating, lack of interest, and fear) and one full scale score. The self-report NIAS will be administered to participants ages 14+, and the parent-report version, the NIAS-PR ([2](#_ENREF_2)), will be completed by parents/guardians of children ages 7-17. *Captures: ARFID-related symptoms.*

Pica, ARFID, and Rumination Disorder Interview-ARFID-Questionnaire (PARDI-AR-Q) ([3](#_ENREF_3)). A 32-item questionnaire derived from the PARDI interview ([4](#_ENREF_4)) that maps on to DSM-5 criteria. One diagnostic prediction (yes/no), one severity score, and three subscale scores (i.e., sensory based avoidance, lack of interest in eating, and concern about aversive consequences) are generated. Both self-report (for ages 14+) and parent report (for parents/guardians of children ages 7-17) will be administered. *Captures: diagnostic prediction, severity of impact, and ARFID-related symptoms.*

***Other Eating Disorder Pathology***

ED100Kv3 ([5](#_ENREF_5)). A self-report instrument based on the Structured Clinical Interview for DSM-5. The ED100Kv3 assesses lifetime AN, BN, BED, and other specified feeding and eating disorders and has been validated among participants ages 12+. The self-report version will be administered to ages 15+. *Captures: Lifetime ED diagnosis, diagnostic transition.*

Eating Disorder Examination-Questionnaire Version 6 (EDE-Qv6) ([6](#_ENREF_6)). A 28-item questionnaire based on the EDE Interview ([6](#_ENREF_6)). Assesses current eating disorder pathology via four subscale scores (i.e., restraint, eating concern, shape concern, and weight concern) and a global score. Participants ages 15+ will complete the self-report version. *Captures: current ED pathology.*

Child Version of the Eight Item Eating Disorder Examination-Questionnaire (ChEDE-Q8) ([7](#_ENREF_7)). An eight-item questionnaire assessing ED pathology in children, validated in children ages 7+. Children ages 7-14 will complete the self-report version. *Captures: current ED pathology.*

Parent Version of the EDE-Qv6 Version 2.0 (PEDE-Qv2.0) ([8](#_ENREF_8)). Adaptation of the EDE-Qv6 for parent report. Items about child’s growth percentile were removed. For parents/guardians of children ages 7-17. *Captures: current ED pathology*

***Depression, Anxiety, and Neurodevelopment***

Patient Health Questionnaire (PHQ-9) ([9](#_ENREF_9)). A well-validated nine-item self-report instrument based on DSM-IV criteria for current major depressive disorder (MDD). Items scored as 0 (not at all) to 3 (nearly every day) are summed to yield a total score. For participants ages 18+. *Captures: current depression severity.*

Patient Health Questionnaire-Adolescents (PHQ-A) ([10](#_ENREF_10)). A self-report version of the PHQ-9 modified for adolescents. Item 9 “Thoughts that you would be better off dead, or of hurting yourself in some way?” was not included. For participants ages 12-17. *Captures: current depression severity.*

Short Mood and Feelings Questionnaire (MFQ) ([11](#_ENREF_11)). A 13-item measure that assesses depressive symptoms during the past two weeks. Items scored 0 (not true) to 2 (true) are summed to yield a total score. All versions can be accessed on the Duke Psychiatry MFQ website ([12](#_ENREF_12)). Three versions will be administered: adult self-report (ages 18+), child self-report (ages 7-17), and parent-report (for parents/guardians of children ages 7-11). *Captures: current depression symptoms.*

Generalized Anxiety Disorder-7 (GAD-7) ([13](#_ENREF_13)). A seven-item self-report measure for generalized anxiety disorder, scored 0 (not at all) to 3 (nearly every day), and summed for a severity score. For participants ages 12+. *Captures: current anxiety symptoms.*

Revised Child Anxiety and Depression Scale (RCADS) ([14](#_ENREF_14)). A 47-item questionnaire assessing DSM anxiety and depressive disorders. Items are on a 4-point scale ranging from 0 (never) to 3 (always) and yield six subscale scores (i.e., separation anxiety disorder, social phobia, generalized anxiety disorder, panic disorder, obsessive-compulsive disorder, and major depressive disorder). For this study, the MDD section was removed, and only five subscale scores are yielded. The self-report child version (RCADS-C) ([14](#_ENREF_14), [15](#_ENREF_15)) will be administered to children ages 7-11. The parent report version (RCADS-P) ([16](#_ENREF_16), [17](#_ENREF_17)) will be given to parents/guardians of children ages 7-11.

Obsessive-Compulsive Inventory-R (OCI-R) ([18](#_ENREF_18)). Assesses OCD-related symptoms with 18 items scored on a 5-point Likert scale ranging from 0 (not at all) to 4 (extremely), summed for a total score. For participants ages 12+. *Captures: OCD symptoms.*

Intellectual/developmental questions. Developed for this study. Parents/guardians of children ages 7-17 will be asked: “Did your child start walking, saying their first words and was potty trained as expected?” “Have you or has a teacher or healthcare professional ever been worried that your child might have difficulties with learning?”, and “Have you ever been told your child has an intellectual disability?” *Captures: intellectual/ developmental disability.*

One Item Temperament Scale ([19](#_ENREF_19)). Parents/guardians of children ages 7-17 will be asked to select one of three statements that best describes the temperament of their children. *Captures: temperament.*

Genetic Links to Anxiety and Depression (GLAD) Study Questionnaire (sections B and C) ([20](#_ENREF_20)). A self-report questionnaire that assesses lifetime MDD and anxiety disorders derived from the UK GLAD study. Modified for this study. For participants ages 15+. *Captures: lifetime MDD and anxiety disorders.*

***Impairment***

Eating Disorder Health-Related Quality of Life (EDQOL) ([21](#_ENREF_21), [22](#_ENREF_22)). A self-report measure assessing health-related quality of life specifically for individuals with an eating disorder. For participants ages 18+. *Captures: ED-related quality of life.*

Short Form Heath Survey (SF-12) ([23](#_ENREF_23)). A widely used 12-item measure assessing self-reported health-related quality of life. Two subscale scores (i.e., physical component and mental component) will be generated. For participants ages 15+. *Captures: general health-related quality of life.*

Pediatric Quality of Life Inventory 4.0 (PedsQL 4.0) ([24](#_ENREF_24)). A 23-item measure assessing health-related quality of life in children and adolescents. Four versions will be administered: child self-report (for children ages 7-12), adolescent self-report (for adolescent ages 13-17), child parent report (for parents/guardians of children ages 7-12), and adolescent parent report (for parents/guardians of adolescents ages 13-17). *Captures: health-related quality of life.*

General health questions. Developed for this study. Items ask about diagnosed neurological problems (e.g., epilepsy), respiratory problems, cardiac problems, structural abnormalities of head and neck, gastroenterological problems, diabetes, celiac disease, or any other medical condition that could affects eating, and food allergies/intolerances. Two versions will be administered: a self-report for participants 18+ and a parent report for parents/guardians of children ages 7-17. *Captures: general health.*

***Environmental Exposures***

Avon Longitudinal Study of Parents and Children (ALSPAC) Major Life Changes (MLC) ([25](#_ENREF_25)). A 22-item self-report checklist that assesses upsetting life events experienced since the age of 12. It is a 22-item measure, but for this study, item 7 was combined with item 8 and item 9 with item 11. Items 14, 16, and 17 will not be asked. Item 21 “Bullying by another person” was slightly modified to “Bullied by another person.” The original questionnaire can be accessed on the ALSPAC website in Section C of “Life of a 16+ Teenager” ([26](#_ENREF_26)). For participants ages 12+. *Captures: childhood environmental exposures.*

Avon Longitudinal Study of Parents and Children (ALSPAC) Upsetting Events (UE) ([25](#_ENREF_25)). A 17-item parent-report checklist that assesses upsetting life events experienced by the child since their 7^th^ birthday. Parents/guardians will have the option of providing a written description for items 1 “My child was taken into care,” 4 “My child had a shock or fright,” and 5 “My child was physically hurt by someone.” The original questionnaire can be accessed on the ALSPAC website in Section E of “My Son/Daughter at Home & at School” ([26](#_ENREF_26)). For parents/guardians of children ages 7-11. *Captures: childhood environmental exposures.*

Pregnancy History Questionnaire (PH) ([27](#_ENREF_27)). Parents/guardians of children ages 7 to 17 will complete the parent-report questionnaire about the mother’s pregnancy history. Adult participants will complete the self-report questionnaire about their mothers’ pregnancy history. *Captures: mother’s pregnancy history.*

Strengths and Difficulties Questionnaire (SDQ) ([28](#_ENREF_28)). A 25-items scored as 0 (not true) to 2 (certainly true). The SDQ assess psychological attributes and yields five subscales (i.e., emotional symptoms, conduct problems, hyperactivity/inattention, peer relationship problems, and prosocial behavior) and a total difficulties score. These questionnaires can be accessed via the youth*in*mind website ([29](#_ENREF_29)). Children ages 12-17 and adults will complete the child and adult single-sided self-report versions. Parents/guardians of children ages 7-11 will complete the single-sided parent report version. *Captures: psychological health.*

***Other***

Free response. Developed for this study. Adult participants will be asked, “Is there anything that we haven't asked that you would like to share about your experience with ARFID or extreme picky/selective eating?” Parents/guardians of children 7-17 will be asked, “Is there anything that we haven't asked that you would like to share about your or your child’s experience with ARFID or extreme picky/selective eating?”

**Aim 2: Within disorder ARFID GWAS.**

We will conduct a comprehensive set of genomic analyses including single nucleotide polymorphism (SNP)-based heritability, GWAS, genetic correlations (r_g_s), and polygenic risk scores (PRS), and standard post-GWAS analyses of ARFID and the non-mutually exclusive ARFID presentations, and rare CNVs and CNV burden.

*a) PGC Ricopili pipeline supports rapid analysis.* A PGC team developed the “Ricopili” software system ([30](#_ENREF_30)) as a robust way to process hundreds of GWAS data sets rapidly and consistently that consists of modules for: pre-imputation (QC), principal components analysis (PCA), imputation, and meta-analysis. We flexibly develop customized pipelines for genetic analysis, using PLINK2 ([31](#_ENREF_31)) or other tools for imputation and genetic analysis ([32](#_ENREF_32), [33](#_ENREF_33)). Ricopili has been used in most primary PGC papers. Briefly, genotype calling uses standard software (with zCall to improve rare variant calling) ([34](#_ENREF_34)). QC and imputation are performed per dataset so that these procedures are performed on technically homogeneous sets of cases and controls. QC parameters for retaining SNPs and subjects: SNP missingness <0.05 (before subject removal); subject missingness <0.02; autosomal heterozygosity deviation (|*F_het_*|<0.2); SNP missingness<0.02 (after subject removal); difference in SNP missingness between cases and controls <0.02; and SNP HWE (*P*>10^−6^ in controls and *P*>10^−10^ in cases). Alternative thresholds can be implemented. Subjects are screened for fingerprinting mismatch, relatedness to any other subject ($\hat{\pi}$<0.2), unusual homozygosity, and sex mismatch. Imputation uses the largest available resources (currently HRC) ([35](#_ENREF_35)) updating to TOPMEd ([36](#_ENREF_36)) (N=65K 30x WGS). Where possible, we will use imputation servers (U Michigan or Sanger). ChrX imputation and analysis is fully implemented (excluding chrX SNPs with missingness ≥ 0.05 or HWE *P*<10^-6^ in females, imputation done separately by sex). The PGC central analysis team supports other analyses including runs of homozygosity ([37-39](#_ENREF_37)), segmental sharing of IBD regions ([40](#_ENREF_40), [41](#_ENREF_41)), gene-by-gene interactions, and conditional analyses as complementary ways to identify loci. Summary statistics are freely disseminated via the PGC website.

Ancestry will be assessed using PCA for each subject, mapped relative to reference samples of known ancestry (Illumina GWAS on 1000 Genomes EUR, AFR, EAS, SAS, and AMR samples ([42](#_ENREF_42)) plus Genome-EUTWIN). Common variants are relatively old; thus, trait associations are expected largely to be shared across global ancestries. Population-specific linkage disequilibrium (LD) patterns that result from drift or natural selection mean that cross-ancestry analyses can help fine-map associated loci. Estimated cross-ancestry genetic correlations are >0.75 (SCZ) for other common diseases ([43](#_ENREF_43)). The PGC has found that the r_g_ for schizophrenia in EUR and East Asian samples was indistinguishable from 1 (.98, se .03). Consistent with our intention to include non-EUR ancestries, we will use established PGC cross-ancestry analytical approaches. The exact method depends on the ancestries represented in the final data and the extent of admixture. We will have direct access to primary genotypes; thus, we can apply mixed modelling approaches using genetic relationship matrix (GRMs) ([44](#_ENREF_44)) as implemented in GCTA and other packages. More trans-ancestry analysis methods are expected to be developed as larger mixed ancestry data sets become available, building upon trans-ancestry meta-analysis ([45](#_ENREF_45)) trans-ancestry LD score regression, the POPCORN method ([46](#_ENREF_46)). The different LD architectures underpin these approaches. If we were to do these analyses now, to account for genetically diverse data with potential hidden structures, we would use a general mixed model (BOLT-LMM) ([47](#_ENREF_47)), supplemented, if necessary, with the big-K, small-K matrices (that account for closer and more distant genetic relationships) ([48](#_ENREF_48)). The exact approach we use will be determined at the time of analysis in consultation with the analysis team and PGC colleagues*.*

*b) Post-GWAS analyses.* The following outlines standard analytic strategy for post-GWAS analysis to maximize information yield and interpretability. The field and methodology evolve rapidly. Below represents what we would do today; however, novel proven methods may emerge before analyses are conducted.

Analysis of chrX. ChrX variants in the pseudo-autosomal regions will be handled separately. SNPs with MAF>0.01 and INFO>0.70 will be retained.

Females and Males. We will conduct secondary GWAS analysis separately on females and males to determine similarity of the results to the primary combined GWAS. As these GWAS may be underpowered for sex-specific differences in genetic risk load, we will calculate PRS (see below) trained on the larger sex sample only and apply it to the smaller sex sample-only target cohort to predict affected status.

Clumping. GWAS results implicate genomic regions (“loci”). To define a locus, SNPs with *P*<5×10^-8^ will be identified and “clumping” will be used to convert significant SNPs to regions. The SNP with the smallest *p* value in a genomic window will be retained as the index SNP and SNPs in LD with the index SNP will define the left and right end of the locus (SNPs with *p*<0.0001 and *r* ^2^>0.1 within 3 Mb windows). Overlapping clumps within 50Kb will be merged into one locus. Conditional analyses ensure candidate loci are statistically independent.

Conditional and joint analysis. Conditional and joint analysis will be conducted using genome-wide complex trait analysis-conditional and joint analysis (GCTA-COJO) ([44](#_ENREF_44)). GCTA-COJO investigates every locus with a joint combination of independent markers via a genome-wide SNP selection procedure. It accounts for LD correlations between SNPs and runs a conditional and joint analysis on the basis of conditional *p* values. After a model optimizing process, the joint effects of all selected SNPs will be calculated. We presume that the ARFID-GEN sample GWAS will be our largest cohort and will be used to approximate the underlying LD structure of the investigated lead SNPs. The conditional regression will be performed in a stepwise manner using the GCTA software ([44](#_ENREF_44)).

Annotation. Genome-wide significant loci are efficiently annotated with FUMA ([49](#_ENREF_49)).

Functional genomic integration. We now routinely use functional genomic results from CommonMind, PsychENCODE, and other efforts to understand GWAS results ([50](#_ENREF_50)). Much of this is automated in FUMA ([49](#_ENREF_49)). We also integrate brain single cell RNA-seq data to identify the cell types implied by the GWAS results. See the PGC MDD paper for examples ([51](#_ENREF_51)).

SNP-based heritability. Linkage disequilibrium score regression (LDSC) will be used to estimate SNP-based heritabilities for ARFID and related presentations ([52](#_ENREF_52), [53](#_ENREF_53)). We will use precomputed LD scores based on the 1000 Genomes Project EUR and ASI ancestry samples ([42](#_ENREF_42)).

PRS. GWAS genotypes will be used to calculate individual PRS. We will use PRS-CS ([54](#_ENREF_54)), a Bayesian polygenic prediction method that infers posterior effect sizes of SNPs using GWAS summary statistics and an external linkage disequilibrium reference panel. Recent studies have shown that PRS-CS leads to enhanced prediction accuracy compared to older PRS methods that are based on arbitrary p-value thresholds and thus discard information. For interpretability, PRS are scaled to represent increased risk and standardized to mean 0 and SD 1. We exclude uncommon SNPs (MAF <0.01), low-quality variants (imputation INFO <0.7), indels, strand-ambiguous SNPs, and SNPs in the extended MHC region (chr6:25-34 Mb).

ARFID presentation analysis. ARFID presentations are overlapping. We will examine the association between ARFID PRS and: a) PARDI-AR-Q subscale scores (sensory sensitivity, phobic avoidance, and low interest/appetite) as continuous scores; and b) a variable categorizing individuals as having a predominant ARFID presentation (e.g., sensory vs. phobic vs. low interest/appetite) defined as highest subscale score.

Gene-wise analysis. MAGMA ([55](#_ENREF_55)) will be used to perform gene-wise tests of association with ARFID based on GWAS summary statistics. MAGMA generates gene-based *p* values by combining SNP-based *p* values within a gene while accounting for LD. To include regulatory regions, SNPs are mapped to genes within 35 kb upstream and 10 kb downstream, and the gene *p* value is obtained using the “multi=snp-wise” model, which aggregates mean and top SNP association models.

Partitioned heritability*.* Partitioned heritability will be investigated using stratified LDSC ([56](#_ENREF_56)), which estimates the per-SNP contribution to overall SNP-heritability (SNP-*h*^2^) across functional annotation categories. It accounts for linked markers and uses a ‘full baseline model’ of annotations that are not specific to any cell type. We will exclude the MHC region in analyses. SNP-*h*^2^ can be partitioned in two ways: a non-cell type-specific and a cell type-specific manner. Many use a somewhat odd set of cell types: we now apply published and replicated biologically-based approaches that leverage single-cell RNAseq data from CNS and PNS from human and mouse. The method fully described in Skene et al. ([57](#_ENREF_57)) and is now routinely used in the PGC.

Gene expression. We will investigate whether ARFID heritability is enriched in tissue/cell type specifically expressed genes using publicly available gene expression data: GTEx ([58](#_ENREF_58)) (RNA-seq of macroscopic samples from multiple human tissues) and increasingly detailed mouse CNS and PNS data (e.g., from colleagues) as part of Cell Atlas. Stratified LDSC estimated common variant heritability enrichment in the top 10% of specifically expressed genes in each tissue or cell type, accounting for confounders such as gene size, LD, and functionally enriched genomic regions (e.g., conserved regions across mammals) ([59](#_ENREF_59)). We will continue to apply published methods (e.g., LDSC-SEG ([60](#_ENREF_60)) as applied to specifically expressed genes) ([58](#_ENREF_58)). From the datasets, the method has derived a genome annotation corresponding to each tissue or cell type of interest, which contains the top 10% specifically expressed genes of the tissue or cell type together with 100 Kb windows on each side of the transcribed region of each genes. Second, we will identify the specific cell types implied by the GWAS results, leveraging the large amount of single-cell and single-nuclei RNA-seq being generated as part of Human Cell Atlas. The method is fully described in Skene et al. ([57](#_ENREF_57)). This area is rapidly evolving and we are active participants (e.g., we have replicated the Skene et al. ([57](#_ENREF_57)) in two new mouse datasets and a deep human cortical dataset from our collaborators at Allen Brain).

Predicted tissue-specific gene expression. We will predict differential gene expression using S-PrediXcan v1.0 ([61](#_ENREF_61)) and genomic and transcriptomic reference data from the brain regions assayed in CommonMind, GTEx v7([58](#_ENREF_58)) and Depression Genes and Networks (DGN) whole-blood cohort ([62](#_ENREF_62)). Significant genes will be compared to genes in the gene-wise analysis performed with MAGMA.

Pathway analyses. We will evaluate whether genes associated with ARFID are enriched in specific pathways, tissues, or cell types. To do this, we will use FUMA to annotate SNPs, identify independent loci, perform pathway analysis, and integrate with a wide array of functional genomic data including gene expression, single cell gene expression, and all available brain epigenetic information. Of particular interest, we will use “SynGO” <https://www.syngoportal.org/> ([63](#_ENREF_63)), a reannotation of synaptic genes by expert synaptic neurobiologists (there are important difference from the standard GO synapse annotations from the literature).

*c) Discover structural variation associated with ARFID*. The PGC CNV working group is actively optimizing CNV calling using GSA data and we will follow these developments closely. Since probe intensity data are the primary input, CNV calling is sensitive to several technical parameters (e.g., batch effects). We will maximize comparability between cases and controls by applying rigorous QC ([64-66](#_ENREF_64)). Following QC, we will fit a series of linear models with CNV burden (at different size thresholds) as the dependent variable to investigate both disease status and experimental biases that can potentially confound CNV detection. A stratified permutation procedure will permute the case-control label within potential confounding factors. Since many known neuropsychiatric CNVs are pleiotropic, we will also test for a global burden of neuropsychiatric CNVs ([67](#_ENREF_67)) in ARFID relative to controls. Following burden tests, we will combine CNV data at the gene/exon level using Ensembl gene models ([68](#_ENREF_68)) and conduct gene/exon level association testing in PLINK ([31](#_ENREF_31)). For each gene/exon, the association test compares CNV counts between cases and controls and uses permutation to determine statistical significance. For each gene/exon, we will annotate the following information: whether this gene has previously been implicated in any prior psychiatric genetic study (CNV, WES, GWAS) ([69](#_ENREF_69)), presence of gene/exon disrupting CNVs, counts of case/control CNVs, and strength of association with ARFID. We will develop a composite score to measure the collective confidence of the copy number change.

**Aim 3. Genetic relation of ARFID to other eating disorders.**

*Analytic plan. a) Disorder-specific GWAS.* We will conduct disorder-specific GWAS for ARFID (and AN, BN, and BED as part of other projects) combining ARFID-GEN with EDGI and existing PGC-ED data using imputed variant dosages and an additive model. Covariates nominally associated with the phenotype in univariate analysis (*p* < 0.05) and five ancestry principal components will be included in GWAS. All cohorts will be meta-analyzed with an inverse-variance weighted fixed-effect model. We are likely to filter our GWAS results with minor allele frequency (MAF) ≥ 0.01 and INFO score ≥ 0.70 (indicating “high-quality”). We anticipate aggregate sample sizes for the PGC-ED by ~2025 (when our collection would be complete) of: (Projected: ARFID=3,000, AN=50,000, BN=20,000, BED=20,000, ARFID-GEN controls [sourced from existing databases]=42,439, PGC-ED controls=543,967). GWAS meta-analysis will be conducted with *ARFID-GEN* and any other ARFID samples that may be introduced to the PGC-ED by time of analysis. To account for genetically diverse data with potential hidden structures and case-control, we will use a general mixed model (BOLT-LMM) ([47](#_ENREF_47)), and if necessary, use big-K/small-K matrices (that account for closer and more distant genetic relationships) ([48](#_ENREF_48)).

*Combined eating disorder GWAS.* Combining *ARFID-GEN* with PGC-ED GWAS of AN, BN, and BED (which will be available by the time our data are analysis-ready), conduct GWAS meta-analysis of all EDs and of component behaviors that cross-cut disorders (e.g., binge eating, restriction), increasing power to identify genetic risk factors that are common across the four disorders.

*b) Genetic correlations:* Common variant-based genetic correlations (SNP-*r*_g_) measure the extent to which two traits or disorders share common genetic variation. We will calculate SNP-*r*_g_ for ARFID, and selected traits using GWAS summary statistics via an analytical extension of LDSC ([52](#_ENREF_52), [53](#_ENREF_53)), as well as explore genome-based restricted maximum likelihood (GREML), which was recently shown to have higher accuracy than LDSC ([70](#_ENREF_70)). We expect considerable advances in available summary statistics from highly powered samples by the time this analysis occurs. Risk factors classically considered as environmental, such as exercise for AN, are now known to be complex traits underpinned by genetic and non-genetic factors. Large community cohorts like UK Biobank, generate GWAS summary statistics for a plethora of phenotypes (e.g., diet, medication, blood metabolites). It is very fast to estimate r_g_ from GWAS summary statistics, and this screening process has the potential to identify unknown associations which will guide the hypotheses of down-stream analyses.

*Generalized summary data-based Mendelian randomization (GSMR)*. Guided by the *r*_g_ estimated above and our hypotheses, we will perform bidirectional Mendelian randomization (MR) analyses to investigate causal relationships between correlated traits and ARFID. For example, it is reasonable to test hypotheses of causality using anxiety as an exposure for ARFID. Significant MR results must be reviewed with caution with respect to strong conclusions about causality as unmeasured confounders may exist. Nonetheless, these analyses are potentially exceptionally informative. Indeed, null MR results are important as they can point at irrelevant or incorrect hypotheses. MR analyses take SNPs that are genome-wide significant for one trait (the exposure) and test the correlation in effect sizes in a second trait (the outcome). Under pleiotropy, there is an expectation that the mean effect size in the outcome trait is different from zero, but under causality a directional relationship in effect sizes is expected. Different versions of MR analysis are highly related; at the time of analysis, we will implement best practices for MR. If we were to do these analyses now, we would use GSMR ([71](#_ENREF_71)) in GCTA ([72](#_ENREF_72)) as it gains a small amount of power over other MR methods by accounting for errors in the estimates of the SNP-exposure trait effect sizes, and in residual correlation between SNPs. We remove potentially pleiotropic SNPs with the heterogeneity in dependent instruments (HEIDI) outlier method ([71](#_ENREF_71)) (pleiotropic SNPs show an effect on the outcome that significantly diverges from that expected under a causal model). Outlier pleiotropy (e.g., as for Apolipoprotein E4 (*APOE4*) between Alzheimer’s Disease and low density lipids cholesterol driven by lipid metabolism) can occur in the context of general pleiotropy, causality, or null association. Pleiotropy is when a single locus directly affects several phenotypes. The method uses the intercept of the bivariate LDSC to account for potential sample overlap ([52](#_ENREF_52), [53](#_ENREF_53)). As a rule of thumb, GSMR requires ≥10 instruments (independent genome-wide significant associations). If necessary, we will use weaker SNP instruments depending on results of the meta-analysis, and interpret results with the appropriate degree of caution. MR can also be used within EDs although causal hypotheses are not straightforward. When MR has been applied between other psychiatric disorders [e.g., MDD-schizophrenia (SCZ)] ([51](#_ENREF_51)), the observed bidirectional significance suggests pleiotropy at the level of shared pathways. MR analyses can be conducted with GWAS summary statistics and be robust to sample overlap.

*Multi-trait-based conditional and joint analysis*. Results from a) and b) will inform these analyses. For example, we expect to detect genetic correlations of BMI with EDs, and MR analyses will aid interpretation of correlation by causality. We will conduct conditional GWAS analyses to determine if the detected SNP associations for EDs can be explained through their relationship with correlated traits. We will perform a multi-trait-based conditional and joint analysis (GCTA-mtCOJO) ([71](#_ENREF_71)) using an extension of GCTA ([72](#_ENREF_72)). This method uses summary-level data to perform conditional analyses. Based on our previous work, we expect to condition the results of our ARFID GWAS on the best available GWAS results for relevant traits including IQ, education years, type 2 diabetes, high density lipid cholesterol, BMI, SCZ, MDD, ASD, OCD, and neuroticism. We will use *ARFID-GEN* individual-level genotype data to approximate the underlying LD structure. As a first step, the method performs GSMR (described above) analysis to test for causal association between the outcome (i.e., ARFID) and the risk factor (e.g., autism). We will remove potentially pleiotropic SNPs using the HEIDI outlier method (see above) ([71](#_ENREF_71)). Second, the GCTA-mtCOJO calculates the genetic correlation between the exposure and the outcome using LDSC to adjust for genetic overlap ([52](#_ENREF_52), [53](#_ENREF_53)). As a result, GCTA-mtCOJO calculates conditional effect sizes, conditional SEs, and conditional *P* values. Subsequently, we will clump the conditional GWAS results using the standard PLINK v1.9 ([73](#_ENREF_73)) algorithm (see above for details) to investigate if any of the genome-wide significant loci showed dependency on genetic variation associated with other phenotypes. GCTA-mtCOJO GWAS results can be used in bivariate LDSC regression. Comparison of these correlations compared to those generated from unconditional GWAS results (i.e., from *a* above) provides insights into the forces shaping the shared genetic relationships between disorders.

*Disorder-specific SNP associations*. A key question in ED research is to understand the differences as well as the similarities among the EDs. This question is very similar to the one posed by PGC colleagues interested in understanding the similarity/differences between SCZ and bipolar disorder (BIP) ([74](#_ENREF_74)). Guided by their analyses, we will conduct case vs. case (e.g., ARFID-AN) GWAS. This approach is powerful if case samples can be grouped (e.g., if genotyped together so that technical confounding factors are not present), as sampling errors associated with control allele frequency estimates are avoided. For SCZ-BIP, additional disorder-specific association results were achieved through using a summary statistics approach (mtCOJO) from standard SCZ vs. control and BIP vs. control analyses, since the loss in power from use of control data was outweighed by the increase in sample size afforded by the approach (as less than 75% of available case samples could be matched into suitable case vs case cohorts). For *ARFID-GEN*, at the time of analysis, we will evaluate the best methodology available to identify ED-specific variants. Disorder-specific analyses are limited by differential power between disorders (e.g., the SCZ GWAS now has greater power than BIP). Differential power will be considered in our interpretation of the analyses in proposed in this sub-aim.

*Multi-PRS (MPS)*. To gain insight into factors underlying ED heterogeneity, we will use the largest available GWAS summary statistics for psychiatric and somatic disorders/traits and combine derived PRS into MPS to predict target outcomes (ARFID, AN, BN, or BED diagnosis), and then more granular phenotypes (e.g., age of onset, severity, low weight). Combining *ARFID-GEN* with EDGI and existing AN, BN, and BED cohorts in the PGC-ED samples should yield a sample size of ~95,000 ED cases and >500,000 controls. Cases may have mixed ancestry, perhaps with greater heterogeneity in some eating disorders. PRS aggregate the effects of many trait-associated genetic variants discovered in GWAS to estimate individual genetic propensities ([75](#_ENREF_75)). As detailed by Kraphol et al. ([75](#_ENREF_75)), we will increase power by using MPS to combine the predictive power of several PRS in one regression model. Training data will combine the best available GWAS summary statistics on psychiatric, metabolic, BMI, anthropometric, personality, physical activity, and educational phenotypes from: PGC colleagues; immediate colleagues working with UK Biobank, Genetic Investigation of Anthropometric Traits (GIANT), the Social Science Genetic Association Consortium (SSGAC), and other consortia; and from published studies. These PRS (normally distributed, 1 df) will be used as genetic predictors in models of ARFID, AN, BN, BED, and more granular phenotypes. This approach is ideal for our overarching intentions of predicting outcomes rather than discovering their etiology (i.e., using not finding genes). MPS is valuable when trait prediction is a priority.

*c) Genomic SEM (GSEM).* We will use lifetime ARFID, AN, BN, and BED GWAS summary statistics. We will employ GSEM ([76](#_ENREF_76)) to identify genetic factors for ARFID and associated EDs. GSEM is a multivariate method for analyzing the joint genetic architecture of complex traits. By modeling covariance structure, GSEM synthesizes genetic correlations and SNP heritabilities inferred from GWAS summary statistics of individual traits from samples with varying and unknown degrees of overlap. GSEM analyses will include several steps:

1. Factor analysis of correlated traits: To better understand and define the genetic factor structure of ARFID and other EDs, we will use GWAS summary statistics and follow exploratory genomic factor analytic (EFA) steps, as recommended: 1) combine files, 2) run multivariable LDSC regression to obtain the genetic covariance and sampling covariance matrices, 3) smooth the genetic covariance matrix for EFA, 4) run EFA, 5) remove traits that do not load well onto any factor, and 6) identify an appropriate factor structure. Based on EFA results, we will fit a model using confirmatory analysis and fine-tune the model (e.g., compare models with correlated vs. uncorrelated factors, confirm number of factors) through statistical comparison of model fit. 2. Estimation of SNP effects: Once a model is confirmed, we will examine specific SNP effects using the GSEM package. Effects for each SNP will be estimated for each model factor. We will also examine heterogeneity statistics (Q_SNP_) by specifying a model in which each SNP has a direct effect on each individual trait in the model and comparing model fit statistics from this model to one in which the SNP effect is mediated by the broad factor(s). We then identify SNPs that show direct effects on specific traits along with those associated with broad factors and compare these results with previously identified loci identified in single-trait PRS. 3. Computation of factor-level PRS: We will save factor-level SNP effects from the final model for development of factor-level PRSs. SNPs will be pruned for LD and factor-level PRS tuned and applied. Prep of the discovery data will involve selecting high-quality autosomal SNPs (INFO ≥0.9; MAF >5%), pruning to remove SNPs in high LD (r^2^ <0.25 in 500kb windows), and removal of major histocompatibility (MHC) region due to high LD structure. We will consult PGC statistical genetics colleagues and update methods accordingly.

**Aim 4. Genetic relation of ARFID with psychiatric, metabolic/anthropometric, neurodevelopmental, and other relevant phenotypes.** Analyses will parallel Aim 3 only with an outward focus on traits other than EDs. For r_g_s, we will follow methods applied in the ANGI-PGC-ED Freeze 2 analysis ([77](#_ENREF_77)), adding additional traits as GWAS summary statistics become available. Only GWAS summary statistics are needed for GSEM, many of which are publicly available. Phenotypes of interest are not directly evaluated in the target sample. Related traits for GSEM may include: lifetime ANX, neuroticism, BMI, fat mass, and fat-free mass (available in the UK Biobank); autism, ADHD, post-traumatic stress disorder (PTSD), MDD, and OCD GWAS summary statistics (available from the PGC).

References

1. Burton Murray H, Dreier MJ, Zickgraf HF, Becker KR, Breithaupt L, Eddy KT, et al. Validation of the nine item ARFID screen (NIAS) subscales for distinguishing ARFID presentations and screening for ARFID. Int J Eat Disord. 2021;54(10):1782-92.

2. Ziolkowska B, Ocalewski J, Zickgraf H, Brytek-Matera A. The Polish Version of the Avoidant/Restrictive Food Intake Disorder Questionnaire-Parents Report (ARFID-Q-PR) and the Nine Items Avoidant/Restrictive Food Intake Disorder Screen-Parents Report (NIAS-PR): Maternal Perspective. Nutrients. 2022;14(15).

3. Bryant-Waugh R, Stern CM, Dreier MJ, Micali N, Cooke LJ, Kuhnle MC, et al. Preliminary validation of the pica, ARFID and rumination disorder interview ARFID questionnaire (PARDI-AR-Q). J Eat Disord. 2022;10(1):179.

4. Bryant-Waugh R, Micali N, Cooke L, Lawson EA, Eddy KT, Thomas JJ. Development of the Pica, ARFID, and Rumination Disorder Interview, a multi-informant, semi-structured interview of feeding disorders across the lifespan: A pilot study for ages 10-22. Int J Eat Disord. 2019;52(4):378-87.

5. Thornton L, Munn-Chernoff M, Baker J, Juréus A, Parker R, Henders A, et al. The Anorexia Nervosa Genetics Initiative (ANGI): Overview and methods. Contemp Clin Trials. 2018;74:61-9.

6. Fairburn CG, Beglin SJ. Assessment of eating disorders: Interview or self-report questionnaire? Int J Eat Disord. 1994;16(4):363-70.

7. Kliem S, Schmidt R, Vogel M, Hiemisch A, Kiess W, Hilbert A. An 8-item short form of the Eating Disorder Examination-Questionnaire adapted for children (ChEDE-Q8). Int J Eat Disord. 2017;50(6):679-86.

8. Drury CR, Hail L, Rienecke RD, Accurso EC, Coelho JS, Lock J, et al. Psychometric properties of the Parent Eating Disorder Examination Questionnaire. Int J Eat Disord. 2023. on;ine ahead of print doi: 10.1002/eat.23999.

9. Kroencke K, Spitzer R, Williams J. The PHQ-9: validity of a brief depression severity measure [Electronic version]. J Gen Intern Med. 2001;16(9):606-13.

10. Johnson JG, Harris ES, Spitzer RL, Williams JB. The patient health questionnaire for adolescents: validation of an instrument for the assessment of mental disorders among adolescent primary care patients. J Adolesc Health. 2002;30(3):196-204.

11. Angold A, Costello, E.J., Messer, S., Pickles, A., Winder, F., Silver, D., . Mood and Feelings Questionnaire: the development of a short questionnaire for use in epidemiological studies of depression in children and adolescents. Int J Methods Psychiatr Res 1995;5:237-49.

12. Duke University Psychiatry and Behavioral Sciences. Mood and Feelings Questionnaire (MFQ). [https://psychiatry.duke.edu/research/research-programs-areas/assessment-intervention/developmental-epidemiology-instruments-0] Accessed July 18, 2023.

13. Lowe B, Decker O, Muller S, Brahler E, Schellberg D, Herzog W, et al. Validation and standardization of the Generalized Anxiety Disorder Screener (GAD-7) in the general population. Med Care. 2008;46(3):266-74.

14. Chorpita BF, Yim L, Moffitt C, Umemoto LA, Francis SE. Assessment of symptoms of DSM-IV anxiety and depression in children: a revised child anxiety and depression scale. Behav Res Ther. 2000;38(8):835-55.

15. Chorpita BF, Moffitt CE, Gray J. Psychometric properties of the Revised Child Anxiety and Depression Scale in a clinical sample. Behav Res Ther. 2005;43(3):309-22.

16. Ebesutani C, Bernstein A, Nakamura BJ, Chorpita BF, Weisz JR, Research Network on Youth Mental H. A psychometric analysis of the revised child anxiety and depression scale--parent version in a clinical sample. J Abnorm Child Psychol. 2010;38(2):249-60.

17. Ebesutani C, Chorpita BF, Higa-McMillan CK, Nakamura BJ, Regan J, Lynch RE. A psychometric analysis of the Revised Child Anxiety and Depression Scales--parent version in a school sample. J Abnorm Child Psychol. 2011;39(2):173-85.

18. Foa EB, Huppert JD, Leiberg S, Langner R, Kichic R, Hajcak G, et al. The Obsessive-Compulsive Inventory: development and validation of a short version. Psychol Assess. 2002;14(4):485.

19. Sleddens EF, Hughes SO, O'Connor TM, Beltran A, Baranowski JC, Nicklas TA, et al. The Children's Behavior Questionnaire very short scale: psychometric properties and development of a one-item temperament scale. Psychol Rep. 2012;110(1):197-217.

20. GLAD Study [Available from: <https://gladstudy.org.uk>]. Accessed July 18, 2023.

21. Engel S. Health Related Quality of Life and Disordered Eating: Development and Validation of the Eating Disorders Quality of Life Instrument. [Unpublished Dissertation]. Fargo, ND: North Dakota State University; 2003.

22. Engel S, Wittrock D, Crosby R, Wonderlich S, Mitchell J, Kolotkin R. Development and psychometric validation of an eating disorder‐specific health‐related quality of life instrument. Int J Eat Disord. 2006;39(1):62-71.

23. Ware J, Jr., Kosinski M, Keller S. A 12-Item Short-Form Health Survey: construction of scales and preliminary tests of reliability and validity. Med Care. 1996;34:220-33.

24. Varni JW, Seid M, Kurtin PS. PedsQL 4.0: reliability and validity of the Pediatric Quality of Life Inventory version 4.0 generic core scales in healthy and patient populations. Med Care. 2001;39(8):800-12.

25. Golding J, Pembrey M, Jones R, Team AS. ALSPAC--the Avon Longitudinal Study of Parents and Children. I. Study methodology. Paediatr Perinat Epidemiol. 2001;15(1):74-87.

26. ALSPAC Child completed questionnaires [Available from: <https://www.bristol.ac.uk/alspac/researchers/our-data/questionnaires/child-completed-questionnaires/>. Accessed July 18, 2023.

27. Mills-Koonce WR, Willoughby MT, Short SJ, Propper CB. The Brain and Early Experience Study: Protocol for a prospective observational study. JMIR Res Protoc. 2022;11(6):e34854.

28. Goodman R. The Strengths and Difficulties Questionnaire: A Research Note. J Child Psychol Psychiatry. 1997;38:581-6.

29. youthinmind English (USA) [Available from: <https://www.sdqinfo.org/py/sdqinfo/b3.py?language=Englishqz(USA>). Accessed July 19 2023.

30. Lam M, Awasthi S, Watson HJ, Goldstein J, Panagiotaropoulou G, Trubetskoy V, et al. RICOPILI: Rapid Imputation for COnsortias PIpeLIne. Bioinform. 2020;36(3):930-3.

31. Purcell S, Neale B, Todd-Brown K, Thomas L, Ferreira Manuel AR, Bender D, et al. PLINK: A tool set for whole-genome association and population-based linkage analyses. Am J Hum Genet. 2007;81(3):559-75.

32. Price AL, Patterson NJ, Plenge RM, Weinblatt ME, Shadick NA, Reich D. Principal components analysis corrects for stratification in genome-wide association studies. Nat Genet. 2006;38:904-9.

33. Willer C, Li Y, Abecasis G. METAL: fast and efficient meta-analysis of genomewide association scans. Bioinform. 2010;26:2190-1.

34. Goldstein JI, Crenshaw A, Carey J, Grant GB, Maguire J, Fromer M, et al. zCall: a rare variant caller for array-based genotyping: genetics and population analysis. Bioinform. 2012;28(19):2543-5.

35. Consortium tHR. A reference panel of 64,976 haplotypes for genotype imputation. Nat Genet. 2016;48(10):1279-83.

36. Brody J, Morrison A, Bis J, O’Connell J, Brown M, Huffman J, et al. NHLBI Trans-Omics for Precision Medicine (TOPMed) Consortium. Cohorts for Heart and Aging Research in Genomic Epidemiology (CHARGE) Consortium. TOPMed Hematology and Hemostasis Working Group. CHARGE Analysis and Bioinformatics Working Group Analysis commons, a team approach to discovery in a big-data environment for genetic epidemiology. Nat Genet. 2017;49:1560-3.

37. Keller MC, Simonson MA, Ripke S, Neale BM, Gejman PV, Howrigan DP, et al. Runs of homozygosity implicate autozygosity as a schizophrenia risk factor. PLoS Genet. 2012;8(4):e1002656.

38. Lencz T, Lambert C, DeRosse P, Burdick KE, Morgan TV, Kane JM, et al. Runs of homozygosity reveal highly penetrant recessive loci in schizophrenia. Proc Natl Acad Sci U S A. 2007;104(50):19942-7.

39. McQuillan R, Leutenegger AL, Abdel-Rahman R, Franklin CS, Pericic M, Barac-Lauc L, et al. Runs of homozygosity in European populations. Am J Hum Genet. 2008;83(3):359-72.

40. Gusev A, Lowe JK, Stoffel M, Daly MJ, Altshuler D, Breslow JL, et al. Whole population, genome-wide mapping of hidden relatedness. Genome Res. 2009;19(2):318-26.

41. Browning SR, Browning BL. High-resolution detection of identity by descent in unrelated individuals. Am J Hum Genet. 2010;86(4):526-39.

42. Genomes Project C, Abecasis GR, Auton A, Brooks LD, DePristo MA, Durbin RM, et al. An integrated map of genetic variation from 1,092 human genomes. Nature. 2012;491(7422):56-65.

43. Liu JZ, Van Sommeren S, Huang H, Ng SC, Alberts R, Takahashi A, et al. Association analyses identify 38 susceptibility loci for inflammatory bowel disease and highlight shared genetic risk across populations. Nat Genet. 2015;47(9):979-86.

44. Yang J, Loos RJF, Powell JE, Medland SE, Speliotes EK, Chasman DI, et al. FTO genotype is associated with phenotypic variability of body mass index. Nature. 2012;490(7419):267-72.

45. Morris AP. Transethnic meta-analysis of genomewide association studies. Genet Epidemiol. 2011;35(8):809-22.

46. Brown BC, Ye CJ, Price AL, Zaitlen N, Consortium AGENTD. Transethnic genetic-correlation estimates from summary statistics. Am J Hum Genet. 2016;99(1):76-88.

47. Loh P-R, Tucker G, Bulik-Sullivan BK, Vilhjálmsson BJ, Finucane HK, Salem RM, et al. Efficient Bayesian mixed-model analysis increases association power in large cohorts. Nat Genet 2015;47(3):284.

48. Zaitlen N, Kraft P, Patterson N, Pasaniuc B, Bhatia G, Pollack S, et al. Using extended genealogy to estimate components of heritability for 23 quantitative and dichotomous traits. PLoS Genet. 2013;9(5):e1003520.

49. Watanabe K, Taskesen E, Van Bochoven A, Posthuma D. Functional mapping and annotation of genetic associations with FUMA. Nat Commun. 2017;8(1):1-11.

50. Fromer M, Roussos P, Sieberts SK, Johnson JS, Kavanagh DH, Perumal TM, et al. Gene expression elucidates functional impact of polygenic risk for schizophrenia. Nat Neurosci. 2016;19(11):1442-53.

51. Wray N, Ripke S, Mattheisen M, Trzaskowski M, Byrne E, Abdellaoui A, et al. Genome-wide association analyses identify 44 risk variants and refine the genetic architecture of major depression. Nat Genet. 2018;50(5):668.

52. Bulik-Sullivan B, Finucane H, Anttila V, Gusev A, Day F, ReproGen Consortium, et al. An atlas of genetic correlations across human diseases and traits. Nat Genet. 2015;47:1236-41.

53. Bulik-Sullivan BK, Loh PR, Finucane HK, Ripke S, Yang J, Schizophrenia Working Group of the Psychiatric Genomics C, et al. LD Score regression distinguishes confounding from polygenicity in genome-wide association studies. Nat Genet. 2015;47:291-5.

54. Ge T, Chen C-Y, Ni Y, Feng Y-CA, Smoller JW. Polygenic prediction via Bayesian regression and continuous shrinkage priors. Nat Commun. 2019;10(1):1-10.

55. de Leeuw C, Mooij J, Heskes T, Posthuma D. MAGMA: generalized gene-set analysis of GWAS data. PLoS Comput Biol 2015;11(4):e1004219.

56. Finucane H, Bulik-Sullivan B, Gusev A, Trynka G, Reshef Y, Loh P-R, et al. Partitioning heritability by functional annotation using genome-wide association summary statistics. Nat Genet. 2015;47(11):1228-35.

57. Skene NG, Bryois J, Bakken TE, Breen G, Crowley JJ, Gaspar HA, et al. Genetic identification of brain cell types underlying schizophrenia. Nat Genet. 2018;50(6):825-33.

58. GTEx Consortium. Genetic effects on gene expression across human tissues. Nature. 2017;550(7675):204.

59. Lindblad-Toh K, Garber M, Zuk O, Lin M, Parker B, Washietl S, et al. A high-resolution map of human evolutionary constraint using 29 mammals. Nature. 2011;478(7370):476-82.

60. Finucane HK, Reshef YA, Anttila V, Slowikowski K, Gusev A, Byrnes A, et al. Heritability enrichment of specifically expressed genes identifies disease-relevant tissues and cell types. Nat Genet. 2018;50(4):621-9.

61. Barbeira AN, Dickinson SP, Bonazzola R, Zheng J, Wheeler HE, Torres JM, et al. Exploring the phenotypic consequences of tissue specific gene expression variation inferred from GWAS summary statistics. Nat Commun. 2018;9(1):1825.

62. Battle A, Mostafavi S, Zhu X, Potash JB, Weissman MM, McCormick C, et al. Characterizing the genetic basis of transcriptome diversity through RNA-sequencing of 922 individuals. Genome Res. 2014;24(1):14-24.

63. Koopmans F, van Nierop P, Andres-Alonso M, Byrnes A, Cijsouw T, Coba MP, et al. SynGO: An Evidence-Based, Expert-Curated Knowledge Base for the Synapse. Neuron. 2019;103(2):217-34 e4.

64. Szatkiewicz JP, Wang W, Sullivan PF, Wang W, Sun W. Improving detection of copy-number variation by simultaneous bias correction and read-depth segmentation. Nucleic Acids Res. 2013;41(3):1519-32.

65. Szatkiewicz JP, Neale BM, O'Dushlaine C, Fromer M, Goldstein JI, Moran JL, et al. Detecting large copy number variants using exome genotyping arrays in a large Swedish schizophrenia sample. Mol Psychiatry. 2013;18(11):1178-84.

66. Ruderfer DM, Chambert K, Moran J, Talkowski M, Chen ES, Gigek C, et al. Mosaic copy number variation in schizophrenia. Eur J Hum Genet. 2013;21(9):1007-11.

67. Kendall KM, Rees E, Bracher-Smith M, Legge S, Riglin L, Zammit S, et al. Association of Rare Copy Number Variants With Risk of Depression. JAMA Psychiatry. 2019;76(8):818-25.

68. Flicek P, Ahmed I, Amode MR, Barrell D, Beal K, Brent S, et al. Ensembl 2013. Nucleic Acids Res. 2013;41(Database issue):D48-55.

69. Konneker T, Barnes T, Furberg H, Losh M, Bulik CM, Sullivan PF. A searchable database of genetic evidence for psychiatric disorders. Am J Med Genet B Neuropsychiatr Genet. 2008;147B:671-5.

70. Ni G, Zeng J, Revez JA, Wang Y, Zheng Z, Ge T, et al. A comparison of ten polygenic score methods for psychiatric disorders applied across multiple cohorts. Biol Psychiatry. 2021;90(9):611-20.

71. Zhu Z, Zheng Z, Zhang F, Wu Y, Trzaskowski M, Maier R, et al. Causal associations between risk factors and common diseases inferred from GWAS summary data. Nat Commun. 2018;9(1):224.

72. Yang J, Lee SH, Goddard ME, Visscher PM. GCTA: a tool for genome-wide complex trait analysis. Am J Hum Genet. 2011;88(1):76-82.

73. Chang CC, Chow CC, Tellier LC, Vattikuti S, Purcell SM, Lee JJ. Second-generation PLINK: rising to the challenge of larger and richer datasets. Gigasci. 2015;4:7.

74. Ruderfer DM, Ripke S, McQuillin A, Boocock J, Stahl EA, Pavlides JMW, et al. Genomic dissection of bipolar disorder and schizophrenia, including 28 subphenotypes. Cell. 2018;173(7):1705-15. e16.

75. Krapohl E, Patel H, Newhouse S, Curtis CJ, von Stumm S, Dale PS, et al. Multi-polygenic score approach to trait prediction. Mol Psychiatry. 2018;23(5):1368-74.

76. Grotzinger AD, Rhemtulla M, de Vlaming R, Ritchie SJ, Mallard TT, Hill WD, et al. Genomic structural equation modelling provides insights into the multivariate genetic architecture of complex traits. Nat Hum Behav. 2019;3(5):513-25.

77. Watson H, Yilmaz Z, Thornton L, Hübel C, Coleman J, Gaspar H, et al. Genome-wide association study identifies eight risk loci and implicates metabo-psychiatric origins for anorexia nervosa. Nat Genet. 2019;51:1207-14.
